# Star Masses and Star-Planet Distances for Earth-like Habitability

**DOI:** 10.1089/ast.2016.1518

**Published:** 2017-01-01

**Authors:** David Waltham

**Affiliations:** Department of Earth Sciences, Royal Holloway University of London, Egham, UK.

## Abstract

This paper presents statistical estimates for the location and duration of habitable zones (HZs) around stars of different mass. The approach is based upon the assumption that Earth's location, and the Sun's mass, should not be highly atypical of inhabited planets. The results support climate-model-based estimates for the location of the Sun's HZ except models giving a present-day outer-edge beyond 1.64 AU. The statistical approach also demonstrates that there is a habitability issue for stars smaller than 0.65 solar masses since, otherwise, Earth would be an extremely atypical inhabited world. It is difficult to remove this anomaly using the assumption that poor habitability of planets orbiting low-mass stars results from unfavorable radiation regimes either before, or after, their stars enter the main sequence. However, the anomaly is well explained if poor habitability results from tidal locking of planets in the HZs of small stars. The expected host-star mass for planets with intelligent life then has a 95% confidence range of 0.78 *M*_⊙_ < *M* < 1.04 *M*_⊙_, and the range for planets with at least simple life is 0.57 *M*_⊙_ < *M* < 1.64 *M*_⊙_. Key Words: Habitability—Habitable zone—Anthropic—Red dwarfs—Initial mass function. Astrobiology 17, 61–77.

## 1. Introduction

Where are the best places to look for life? This question is usually tackled by building detailed conceptual, mathematical, or computational models of potential habitats to assess their suitability. Lammer *et al.* (2009) gave an excellent and comprehensive review of such climatic, geochemical, and geophysical models together with their predictions concerning the habitability of a variety of worlds. The current paper tackles the same issues in a different way; it uses the fact that Earth is inhabited to statistically constrain properties affecting habitability. The paper considers two properties in particular—the radius of a planet's orbit and the mass of its host star.

At the heart of the paper lie two principles: (i) the Copernican principle that, in the absence of any data to the contrary, we should expect Earth to be reasonably typical; (ii) the Anthropic principle that Earth must possess all properties necessary for the emergence of intelligent observers. A thorough review of the Copernican and Anthropic principles was given by Barrow and Tipler (1986), and they have also been discussed in a number of other books (*e.g.*, Ward and Brownlee, 2000; Scharf, 2014; Waltham, 2014). There is an apparent antagonism between the Copernican and Anthropic principles, but it can be resolved by combining them into the single statement that *Earth is likely to be typical of the subset of planets that possess intelligent observers*. This is close to being tautologically true since, by definition, “typical” is more likely than “atypical.” Nevertheless, the methodology presented below will show that this is a powerful statement that can be used to quantitatively assess factors proposed as important for habitability. Moreover, although this statement implies that conclusions can only be drawn about the habitability requirements for intelligent observers, this paper will show that the results can be extended to give insights into the conditions required for life more generally, albeit only for the case of life in an “Earth-like” habitat (*i.e.*, it gives no insights into other possible habitat types such as the subsurface oceans of icy moons).

The current paper's approach combines Bayes theorem (Hoff, 2009) with Carter's (1983) *n*-step model for the emergence of intelligent observers. Bayes theorem is a statistical technique that tells us “how [our beliefs] should change after seeing new information” (Hoff, 2009). The Carter model assumes that intelligence can only emerge after a series of major evolutionary steps such as the origin of life, the origin of photosynthesis, the origin of eukaryotes, and so on. Together, Bayes theorem plus Carter's model tell us how the probability distribution of a particular planetary property should be modified given the additional information that the planet possesses intelligent life.

The early sections of this paper review the Carter model and show how to combine it with Bayes theorem. The paper then examines estimates for the number of critical steps required for intelligence to evolve. Once this background has been established, the paper investigates the effect on habitability of star-planet separation and star mass.

For brevity, the term *inhabited* is frequently used in the very restrictive sense of denoting planets inhabited by intelligent observers, since this is the focus of the majority of the paper. However, toward the end, the paper addresses how to generalize the results to give probability distributions for life, in general, rather than just intelligent life.

The key result from the paper is the establishment of a new technique for assessing habitability hypotheses. However, in addition, it also gives a clear prediction of which stellar masses should be focused on by SETI and a clear prediction of the slightly different stellar masses that should be the focus for more general searches for life on the surfaces of planets (*e.g.*, searches using spectral biosignatures).

## 2. Probability Distributions for Inhabited Worlds

Probability density functions (pdfs) are central to this paper. A pdf expresses the probability per unit interval for a particular property, for example, the probability of a randomly chosen planet having an age between, say, 3499.5 and 3500.5 million years (strictly speaking, it is defined as the limit of probability ÷ interval as the interval approaches zero). The peak of the distribution indicates the most likely value, and the width of the distribution indicates the range of possible values.

An equally important concept is that of conditional probability, that is, the probability of an event occurring given that some other event has already happened. In the context of this paper, this is relevant because the expected values of planetary properties will be altered if we are given the additional information that the planet is inhabited. Take, for example, the specific case of mean surface temperature, *T*. If life requires liquid water, the conditional probability *p*(*T*/*i*) (*i.e.*, the probability distribution for *T* given that the planet is inhabited) will be nonzero only for a narrow range of temperatures. In contrast, the temperature distribution for all planets, *p*(*T*), will be far broader, as it will include worlds with environments ranging from warmer than Mercury to colder than Pluto.

In principle, the probability distributions for property *x* [*i.e.*, *p*(*x*) and *p*(*x*/*i*)] could be estimated simply by collecting the right data. For example, we could measure the surface temperature of 1000 randomly chosen planets and then the surface temperature of 1000 inhabited planets. However, while it is conceivable that we may soon be able to do the former, we currently know of only one inhabited planet (Earth), so direct construction of pdfs for inhabited planets is unlikely to be possible for the foreseeable future. Instead, Bayes theorem (Hoff, 2009) gives an indirect way to do this by relating the general pdf to the conditional pdf through

\begin{align*}
p \left( {x / i} \right) { \rm{ }} = p \left( x \right)  p \left( {i / x} \right) { \rm{ }} / p \left( i \right) \tag{1}
\end{align*}

Here, *p*(*i*/*x*) [not to be confused with *p*(*x/i*) discussed above] is the probability of intelligence given *x;* that is, *p*(*i*/*x*) is high for some values of *x* and low for others so that this expresses the influence property *x* has on the emergence of intelligence. The final term, *p*(*i*), is a constant that gives the overall probability of intelligence arising on a randomly chosen planet and ensures that Eq. 1 is correctly normalized. Note that, unless *p*(*i*/*x*) is completely flat, *p*(*x*/*i*) will be a different shape to *p*(*x*). Hence, Eq. 1 is a mathematical encapsulation of the anthropic principle that properties of Earth are biased, compared to the general population of planets, for any properties that influence the likelihood of intelligence (Waltham, 2007).

If circumstances are otherwise favorable, the probability of intelligence should monotonically increase with time available; that is, it starts at zero (intelligence is not expected on a planet that is only briefly habitable) and increases to unity given enough time (any event with nonzero probability must happen eventually). Hence, *p*(*i*/*x*) depends upon two factors: (i) how the quality of the habitat is affected by *x;* (ii) how the duration of habitability is affected by *x*.

The effect of habitable duration can be quantified by using insights from Carter (1983). Carter's model for intelligence assumed it required a large number of successive evolutionary steps. These steps were divided into those that are short compared to the time available and those that are long. It was then shown that the time taken for the short steps could be ignored so that the time for intelligence to emerge is controlled by a small number, *n*, of critical, slow steps, steps likely to be associated with major evolutionary transitions such as the origin of life and the origin of eukaryotes. Carter (1983) then showed that the characteristic time for the emergence of intelligence is almost certainly much longer than the characteristic timescale for the evolution of stars since, otherwise, there has been an unlikely coincidence on Earth between the time for intelligence to emerge [a 4-billion-year (4 Gy) timescale governed by biological processes in organisms] and the duration of habitability (a 5 Gy timescale governed by physical processes in stars). If the true timescale for intelligence is actually much longer than the timescale for habitability, then we would expect, in the very rare cases where it manages to emerge at all, that it will do so toward the end of habitability, since appearing earlier is even less likely. Hence, this explanation avoids the need for an unlikely coincidence.

Interestingly, there is a direct analogy between the emergence of intelligence on a habitable planet and the emergence of cancer in an organism. The multistage model of cancer occurrence—the hypothesis that cancers develop only once a cell has undergone several, successive and unlikely (in any given cell) mutations—is similar to the *n*-stage model for the emergence of intelligence. Furthermore, for the case of cancers unlike the case of inhabited planets, we sadly have multiple examples, and these have allowed a mathematically identical model to that of Carter (1983) to be successfully tested using cancer-occurrence statistics [see Nunney (2015) for a review].

From the point of view of the current paper, the most important result that emerges from Carter's (1983) analysis is that the probability of intelligence increases with time according to

\begin{align*}
{ \rm{Probability}} \propto{ {\tau} ^n} \tag{2}
\end{align*}

where τ is the duration of habitability. In the notation of Eq. 1, and taking account of the fact that the probability that intelligence arises also depends upon habitat quality, this can be rewritten as

\begin{align*}
p \left( {i / x} \right) { \rm{ }} = q \left( x \right) \tau { \left( x \right) ^n} \tag{3}
\end{align*}

where *q* quantifies how habitat quality changes with *x* (but see further discussion below). Equations 1 and 3 then combine to yield the central equation of this paper that

\begin{align*}
p \left( {x / i} \right) { \rm{ }} = K \, q \left( x \right) { \rm{ }}p \left( x \right) \tau { \left( x \right) ^n} \tag{4}
\end{align*}

where *K* is a constant found by requiring that the integrated probability is unity.

Habitat quality will, of course, depend upon many factors, and this is not properly accounted for in Eq. 3. For example, *x* might be temperature, as before, but *q* will depend upon other factors such as planetary mass, volatile inventory, and geological activity. However, this paper only considers the effects of one parameter at a time, so an assumption will be made that the planets under consideration are all good habitats apart from the consequences of parameter *x*. I will refer to such worlds, herein, as potentially inhabitable planets.

## 3. How Many Critical Steps?

Before Eq. 4 can be used, we need an estimate of the number of critical steps, *n*. Carter (1983) showed that the critical steps should be roughly equally spaced through time and that, therefore, the time of the final step is

\begin{align*}
{t_n}  \approx  { \rm{ }} \left( {n / n + { \rm{ }}1} \right) \tau \tag{5}
\end{align*}

Carter's own estimate for *n* was unrealistically low as he assumed that Earth would remain habitable throughout the whole of our Sun's main sequence lifetime (*i.e.*, *τ*∼10 Gy), but Watson (2008) used a more reasonable estimate that intelligence has emerged roughly *t_n_* = 4 Gy into a *τ* = 5 Gy habitable lifetime and, hence, *n*∼4.

However, since the Carter (1983) argument is a statistical one, it is also necessary to consider stochastic fluctuations. This can be done by using the expression, derived in Watson (2008), that the pdf for the *m*^th^ step in an *n*-step process is

\begin{align*}
{p_{m / n}} \left( t \right) { \rm{ }} = { \rm{ }} \left[ {n! / \left( {n - m} \right) ! \left( {m - { \rm{ }}1} \right) !} \right] \left[ {{t^m}^{ - 1}{{ \left( { \tau - t} \right) }^n}^{ - m} / { \tau ^n}} \right] \tag{6}
\end{align*}

Taking *m* = *n* and integrating gives the cumulative probability for the timing of the emergence of intelligence as

\begin{align*}
{P_{n / n}} \left( {{t_n}  <  t} \right) { \rm{ }} = { \rm{ }}{ \left( {t / \tau } \right) ^n} \tag{7}
\end{align*}

**FIG. 1.**
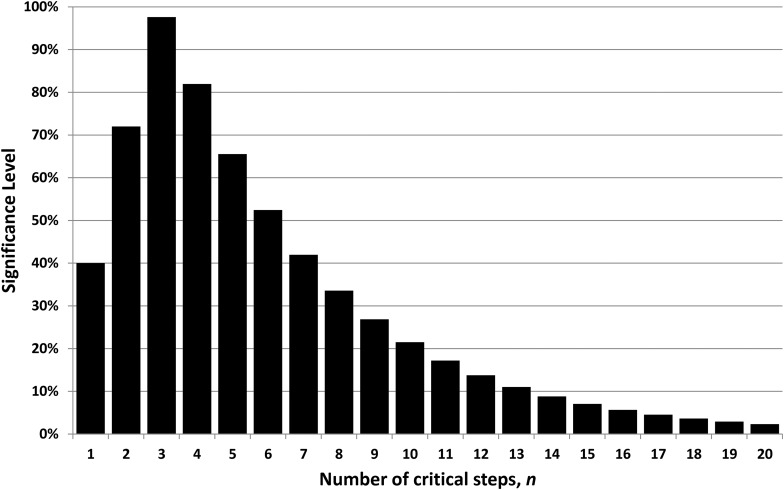
Significance level, for the *n*-step model, constrained by assuming intelligence emerges 4 Gy into a 5 Gy habitable lifespan. This distribution implies a best guess that there are 3 or 4 critical steps, but the significance level exceeds 5% for *n* = 1–16. The number of steps is therefore poorly constrained.

The (two-tailed) significance level is then 2*P_n/n_* (if *P_n/n_* < 0.5) or 2(1 − *P_n/n_*) (if *P_n/n_* > 0.5). This is a measure of how unsurprising the observed timing for intelligence is; that is, significance = 100% is not at all surprising, while significance of 5% (say) indicates a substantial deviation from expectation. Figure 1 plots significance as a function of *n*. The figure shows that the 95% confidence range (*i.e.*, values where significance >5%) extends from *n* = 1 to 16. The number of critical steps is therefore not well constrained by the observed timing for the emergence of intelligence on Earth, although a value around 3 or 4 is most likely.

A further constraint can be introduced by using estimates for the timing of the first step and assuming that this is the origin of life. For that calculation, I assume habitability began when liquid water first appeared [*i.e.*, by 4.4 billion years ago (Ga), Valley *et al.* (2002)]. Unfortunately, estimates for how long it then took life to appear remain highly contentious. Arguments that possible banded iron formations of Isua, Greenland, show isotopic evidence for life at around 3.85 Ga are not universally accepted (*e.g.*, see Moorbath, 2005). However, 3.7 Ga turbidite deposits in the same region show more robust evidence for biogenic alteration in carbon-isotope ratios (Rosing *et al.*, 1996; Fedo *et al.*, 2006), so here, I accept 3.7 Ga as the age of the earliest life so far discovered. This implies that life emerged within 0.7 Gy of the first appearance of water, but this is an estimate that is likely to be subject to much revision in the future. The sensitivity of the results to changes in these timings will therefore be looked at later in this section, but for now, I will proceed using these timings.

**FIG. 2.**
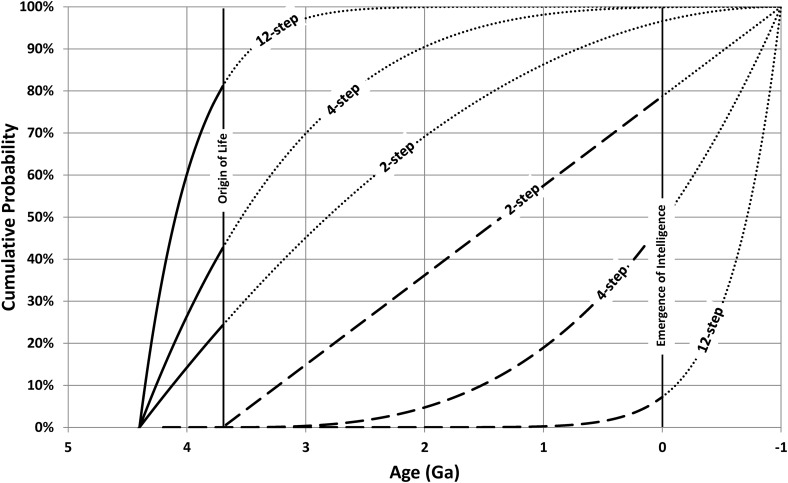
The solid curves (continued with dotted lines) show the cumulative probability for the emergence of life, while the dashed curves (continued with dotted lines) show the cumulative probability for the emergence of intelligence. The vertical lines show the assumed true timing of these events. The 4-step model is an excellent fit (both events occur near a cumulative probability of 0.5), but even the 12-step model is not far enough away from this ideal to be excluded.

Carter's (1983) argument that the critical steps should be, roughly, evenly spaced through Earth's history then gives an origin-of-life-based estimate of *n*∼(5 Gy/0.7 Gy)∼7, which is larger than Watson's (2008) estimate of *n*∼4. However, the two approaches can be combined to yield an improved estimate by regarding the emergence of intelligence as an *n* − 1 step process whose clock begins ticking immediately after the origin of life. Equation 6 can then be used to predict the cumulative probability for the timing of life (integrate *p*_1/*m*_) and for the timing of intelligence (integrate *p_n_*_ − 1/*n* − 1_). Figure 2 shows this for *n* = 2, 4, and 12.

For the 4-step model, the probability that life should have emerged by the assumed time of 3.7 Ga is 43%, while the probability that intelligence emerges by the observed time of 0 Ga is 49%. Both of these figures are close to the median cumulative probability of 50%, so the 4-step model accounts well for both observations. However, even if *n* is as large as 12, the corresponding probabilities have only become 81% and 7%, respectively, and these are still not extreme enough to exclude *n* = 12.

**FIG. 3.**
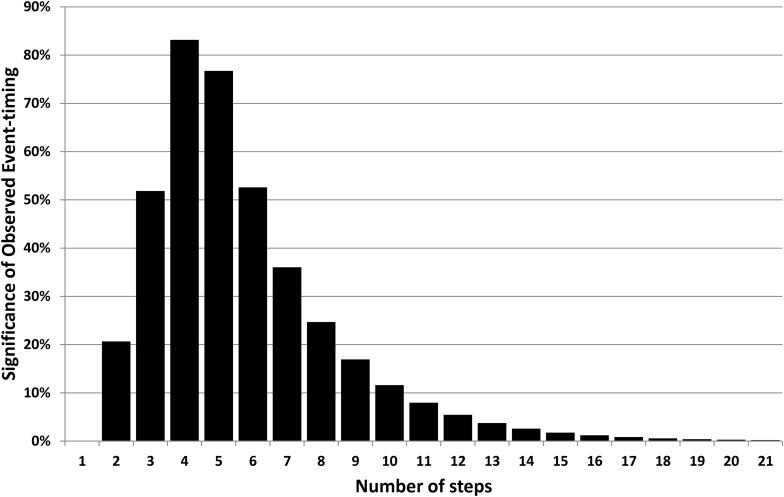
The joint statistical significance of the observed timing for the origin of life and the emergence of intelligence. Models with *n* between 3 and 6 are an excellent fit, but the significance level remains above 5% over the range *n* = 1–12.

As with the analysis illustrated in Fig. 1, the significance level can be calculated for each of these events (origin of life and origin of intelligence); then the additional step can be taken of calculating the significance product. This product is the probability that both events differ from the median by at least as much as observed and can be taken as a joint significance level given the timing of both life and intelligence. This significance is plotted, as a function of *n*, in Fig. 3, which shows that *n* is likely to be in the range 3–6 and is almost certainly 12 or less.

However, as already discussed, the timings of the critical events are themselves poorly constrained, so sensitivity to their uncertainty must also be investigated. Figure 3 can be recalculated by using different assumptions for the timings of the beginning of habitability, the origin of life, and the end of habitability. The biggest changes are produced by assuming that future habitable life span is much smaller (*e.g.*, 0.5 Gy) and that the origin of life was much closer in time to the onset of habitability (*e.g.*, within 0.2 Gy). Such changes push the peak of Fig. 3 up to *n* = 7 and give a much longer tail. At the other extreme, if Earth is assumed to be habitable for another 1.5 Gy and, furthermore, if the origin of life is taken as only being confirmed by the bacterial fossils of the Gunflint Formation at 1.9 Ga, the allowed range shifts down to only *n* = 2–4. It is even possible that the origin of life is not a critical step (or not the first such step) or that intelligence is not the last critical step (*e.g.*, if it is an inevitable result of some earlier innovation), and these issues introduce further uncertainty into the analysis.

The number of critical steps is therefore not well constrained. The remainder of this paper will take *n* = 4 as the best guess but will also look at sensitivity to reasonable changes in this assumption.

## 4. The Sun's Habitable Zone

As an introduction to the use of Eq. 4, this section investigates the location of the Sun's present-day habitable zone (HZ). Published estimates of HZ location are based upon climate model predictions of what would happen to a habitable planet under varying conditions of illumination. This section shows how these model-based estimates can be statistically tested by using the additional constraint that Earth's true location is more likely to be near the middle of the resulting distribution than in its tails. This distribution is, in turn, controlled by the variation in habitable lifetime as planet location is altered; that is, locations that stay within the HZ for a long time are more likely to produce intelligent organisms than locations that are only briefly habitable.

It should be noted that the resulting HZ is not the classic HZ as defined by the range of distances, from a star, where liquid water could be stable on a planetary surface (Huang, 1959). Instead, the HZ is implicitly defined as the range of star-planet separations over which conditions allow operation of the *n*-step process that leads to intelligence. It is plausible to suggest that this *n*-step process can begin once conditions are warm enough for liquid water; hence, the resulting location for the outer edge of the HZ may be identical for the two definitions. The inner edge could be a different matter, since the maximum temperature for metazoan life is probably less than 60°C (Lee, 2003), implying that conditions suitable for intelligent life may end before a planet warms so much that it loses all liquid water. However, the temperature for onset of a runaway moist greenhouse is not much above 60°C (Kasting *et al.*, 1993); hence, the inner edge of the HZ may also not differ very much between the two definitions. In any event, this issue does not affect later conclusions about the effects of star mass on habitability since the HZ obtained in this section is the appropriate one for that analysis.

The starting point for a statistical determination of HZ location is to determine habitable lifetime as a function of star-planet distance, and this requires an evolution model for solar-mass stars. This paper uses the on-line evolution-grids described by Girardi *et al.* (2000) (more specifically, the *Z* = 0.019 grids for masses between 0.6 and 2.0 *M*_⊙_). Other stellar evolution models could be used (*e.g.*, Spada *et al.*, 2013; Valle *et al.*, 2014; Stancliffe *et al.* 2016), but the resulting changes are not significant, as uncertainties in stellar evolution are small compared to issues such as the uncertainty in *n* discussed above.

**FIG. 4.**
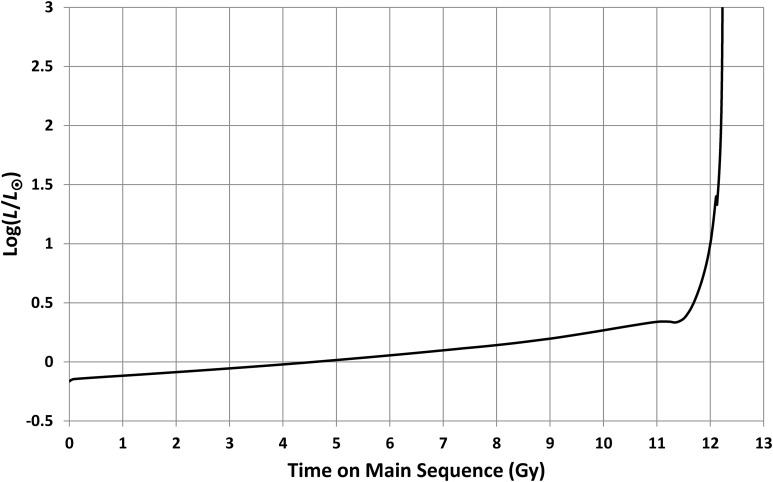
Evolution in luminosity for a solar-mass star (from Girardi *et al.*, 2000). Brightness increases steadily for ∼11 Gy and then jumps by a factor >1000 as the star exhausts its H fuel and leaves the main sequence. *L*_⊙_ is the current solar luminosity.

The evolution in luminosity, *L*, for a Sun-like star is shown in Fig. 4. Zero-age on this graph corresponds to the onset of hydrogen fusion, but the star's brightness then increases slowly for over 11 Gy before increasing dramatically as exhaustion of hydrogen leads to fusion of heavier elements. Note that the ⊙ subscript denotes present-day solar values and will be used throughout this paper.

Assuming that the limits of habitability are controlled by illumination (which is proportional to stellar luminosity and inversely proportional to the square of the star-planet separation), the inner location of the HZ will evolve through time according to

\begin{align*}
{a_{\rm i}} \left( t \right) { \rm{ }} = {a_{{ \rm{i}}0}}{ [ L \left( t \right) / {L_ \odot } ] ^{1 / 2}} \tag{8}
\end{align*}

while the outer location will evolve as

\begin{align*}
{a_{\rm o}} \left( t \right) { \rm{ }} = {a_{{\rm o}0}}{ [ L \left( t \right) / {L_ \odot } ] ^{1 / 2}} \tag{9}
\end{align*}

where *a*_i0_ is the present-day location of the inner boundary of Earth's HZ while *a*_o0_ is the corresponding outer boundary.

**FIG. 5.**
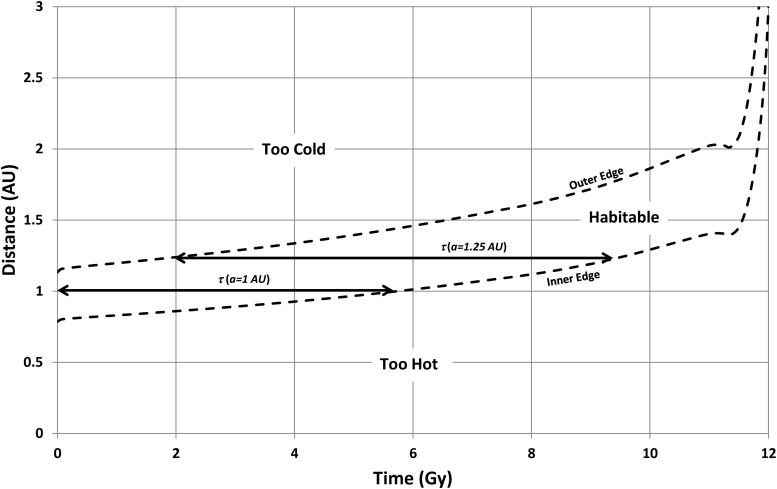
Habitable zone evolution for a solar-mass star. Note that the habitable lifetime, *τ*, changes with star-planet separation (horizontal arrows).

**FIG. 6.**
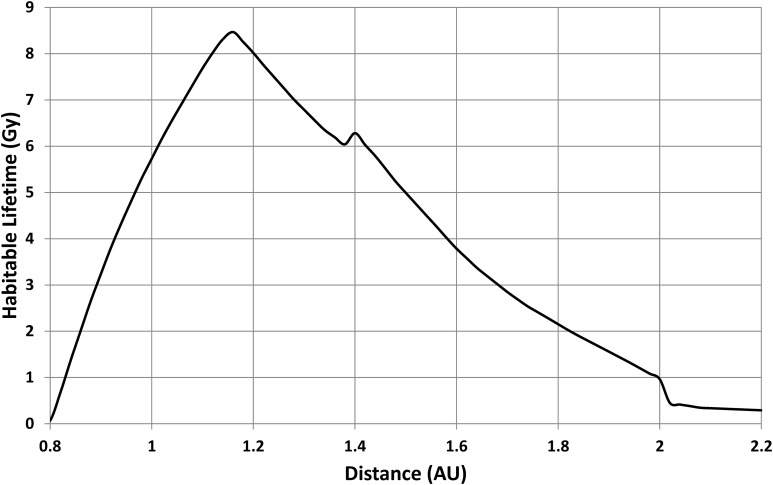
Predicted habitable lifetime from Fig. 5. [Note that the small, additional peak at 1.4 AU is produced by the temporary drop in luminosity seen at the end of the main sequence lifetime (Fig. 4); this produces a jump in the time at which the inner edge of the HZ reaches a planet at 1.4 AU compared to the time when the inner edge reaches a planet slightly closer to the star.]

As an illustrative example, Kasting *et al.'s* (1993) estimate for the present-day HZ (*a*_i0_ = 0.95 AU and *a*_o0_ = 1.37 AU) produces the results shown in Fig. 5. With these HZ limits, planets closer than 0.79 AU are permanently too warm, while planets beyond 2 AU never become warm enough during the main sequence phase. Between these extremes, habitable lifetime gradually increases and then drops again. For example, note that the habitable lifetime at a distance of 1 AU extends from 0 to 5.7 Gy (*i.e.*, a duration of 5.7 Gy), while, at a distance of 1.25 AU, a planet only becomes habitable after ∼2 Gy but remains habitable until ∼9.5 Gy (*i.e.*, a duration of ∼7.5 Gy). The full pattern of change in habitable lifetime with distance is shown in Fig. 6, which shows a peak of 8.5 Gy at 1.16 AU.

Figure 6 is the information needed in Eq. 4 to produce a probability distribution for inhabited planets orbiting solar-mass stars. If the property of interest is star-planet separation, *a*, then Eq. 4 becomes

\begin{align*}
p \left( {a / i} \right) { \rm{ }} = K \ q \left( a \right) { \rm{ }}p \left( a \right) \tau { \left( a \right) ^n} \tag{10}
\end{align*}

with *τ*(*a*) being obtained from Fig. 6.

However, the other component distributions in Eq. 10 will need to be determined. The distribution of potentially inhabitable planets orbiting solar-mass stars, *p*(*a*), can be assumed to be approximately uniform over the relatively narrow width of the HZ. Other reasonable distributions (*e.g.*, logarithmic) give similar results to those shown below. Hence, *p*(*a*) can be subsumed into *K*.

It would be similarly helpful to be able to assume a uniform *q*(*a*), but this is more problematic. Planets relatively close to their star will be potentially habitable earlier than planets farther away (see Fig. 5), so assuming a constant *q*(*a*) implies that the emergence of intelligent life is not affected by the timing of habitability (*e.g.*, 5 Gy of habitability early in a planet's history is as good as 5 Gy of habitability later on). This may not be correct, but to make progress, this paper will assume that this is not an important effect.

**FIG. 7.**
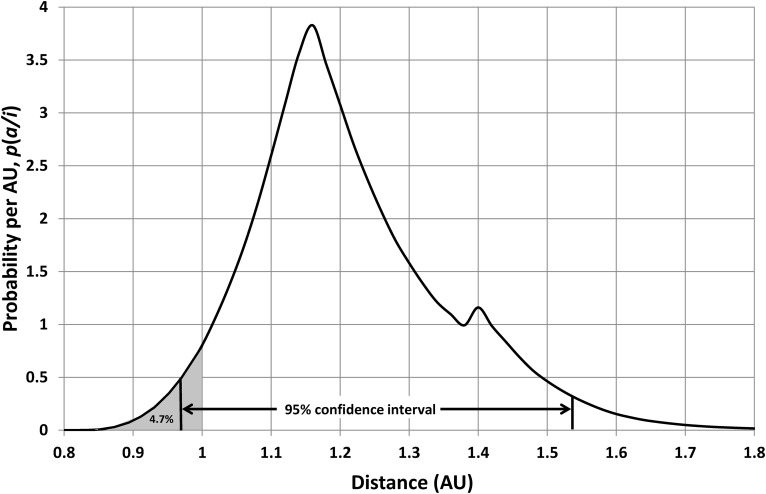
Probability distribution of star-planet separation for planets, with intelligent life, orbiting a solar-mass star. Ninety-five percent of all such planets orbit in the confidence interval 0.97 AU < *a* < 1.54 AU, while 4.7% of all such planets orbit within 1 AU of their star. This distribution assumes *n* = 4 and the Kasting *et al.* (1993) boundaries for Earth's current HZ.

For constant *p*(*a*) and *q*(*a*) along with *n* = 4, Eq. 10 gives the probability shown in Fig. 7. This distribution has a 95% confidence range of 0.97 AU < *a* < 1.54 AU (*i.e.*, 2.5% of the area under the curve is below 0.97 AU, and 2.5% of the area under the curve is above 1.54 AU). Equivalently, there is a cumulative probability of 4.7% that a randomly chosen inhabited planet will have an orbital radius of 1 AU or less, so the cumulative probability for Earth is above 2.5% and below 97.5%. With either formulation, the Kasting *et al.* (1993) HZ hypothesis is accepted at a 5% significance level (strictly speaking, it is not rejected); that is, Kasting *et al.'s* model puts Earth in a reasonably typical location.

The foregoing analysis was dependent upon three parameters: (i) the present-day location of the inner edge of our HZ; (ii) the present-day location of the outer edge of our HZ; (iii) the assumed number of critical steps required for the emergence of intelligence. The analysis could therefore be repeated for other values of these parameters to test other HZ models.

Here, however, the statistical approach will be used to place limits on parameters, rather than to test further, specific hypotheses. In particular, this will be done for *a*_o0_, as the other two parameters are better constrained; there is reasonable consensus over the location of the inner edge of the present-day HZ [Kasting *et al.* (1993) and Franck *et al.* (2000) both used 0.95 AU, the Kopparapu *et al.* (2014) analysis was equivalent to selecting 0.949–0.964 AU, and Hart (1979) suggested nearly four decades ago that *a*_i0_ = 0.958 AU], while the earlier discussion gives confidence that *n*∼3–6. In contrast, there is no general agreement about the outer-edge location, with estimates ranging from 1.2 AU (Franck *et al.*, 2000) to 1.7 AU (Kopparapu *et al.*, 2013) with an absolute limit set as far out as 2.4 AU (Mischna *et al.*, 2000). Hence, the location of *a*_o0_ is the most interesting and useful parameter to statistically constrain.

A lower bound to *a*_o0_ can be found from Eq. 9 together with the constraint that the outer edge of the HZ must have been >1 AU when liquid water first appeared on Earth. Taking, as before, an estimate that this occurred around 0.2 Gy after the origin of Earth gives *L*(*t*)*/L*_⊙_∼0.72 (see Fig. 4) and hence *a*_o0_ > 1.18 AU.

**FIG. 8.**
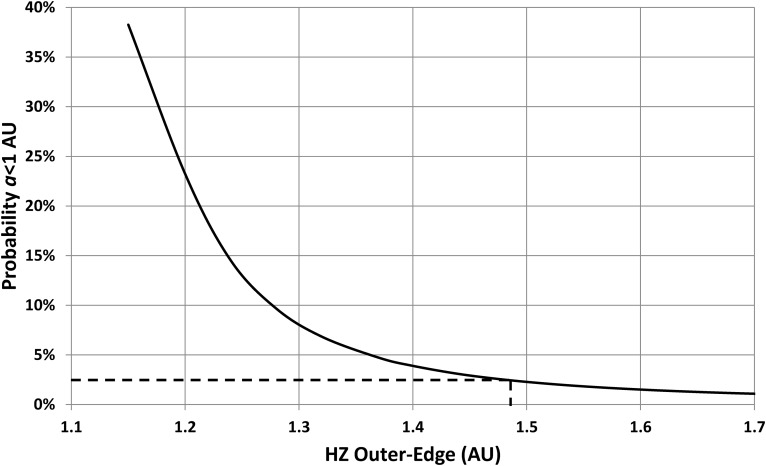
Probability that an inhabited planet orbits within 1 AU of a solar-mass star, as a function of assumed distance to the outer edge of the HZ. Outer-edge distances greater than 1.48 AU would imply that Earth's orbit is surprisingly small (*i.e.*, happens to less than 2.5% of all inhabited planets). Hence, 1.48 AU is an upper limit for the outer edge of Earth's HZ.

To obtain an upper bound for this parameter, the calculations used to produce Fig. 7 were repeated over a range of values for *a*_o0_. The resulting cumulative-probability dependence is shown in Fig. 8, which shows that this falls with increasing *a*_o0_ and reaches 2.5% at *a*_o0_ = 1.48 AU. This is therefore an estimate of an upper limit for the HZ outer edge, since choosing values larger than this puts Earth closer to the Sun than all but 2.5% of inhabited planets; that is, larger values for *a*_o0_ make Earth look like an outlier rather than a typical inhabited world.

However, account must also be taken of the fact that the uncertainties in *n* may produce large changes in the predicted upper bound for *a*_o0_. Taking 3 ≤ *n* ≤ 6 gives *a*_o0_ < 1.50 ± 0.14 AU. The final result is therefore that the outer edge of the Sun's present-day HZ is likely to be in the range 1.18 AU < *a*_o0_ < 1.50 ± 0.14 AU. This statistically derived result suggests that some of the higher climate-model-derived estimates are too large and that models that predict an HZ outer edge beyond ∼1.64 AU should be viewed with caution.

The remainder of this paper will use the Kasting *et al.* (1993) estimate that *a*_o0_ = 1.37 AU, since this sits near the center of the statistically derived range. However, changes in this value do not substantially alter the paper's later conclusions.

## 5. Habitable Lifetime as a Function of Star Mass

The next stages in this paper's analysis require estimates of how the *typical* habitable lifetime and habitable star-planet distance change with stellar mass. The probability-weighted mean values are the obvious estimates to use and are given by

\begin{align*}
\bar \tau & = \int_0^{\infty} {p \tau \, da} {/} \int_0^{\infty}
{p \, da} \\ & = \int_0^{\infty}{\tau^{n + 1} \, da} {/}
\int_0^{\infty} {\tau^n \, da}
 \tag{11} \end{align*}

and

\begin{align*}
\bar a  = \int_0^{\infty}{pa\,da}{/} \int_0^{\infty} {p \, da}
\\  = \int_0^{\infty} {\tau^n}a \, da {/}
\int_0^{\infty}{\tau^n \,da}  \tag{12}
\end{align*}

For a solar-mass star, the lifetime distribution shown in Fig. 6 then gives a mean habitable lifetime of 7.1 Gy and a mean star-planet separation of 1.21 AU.

**FIG. 9.**
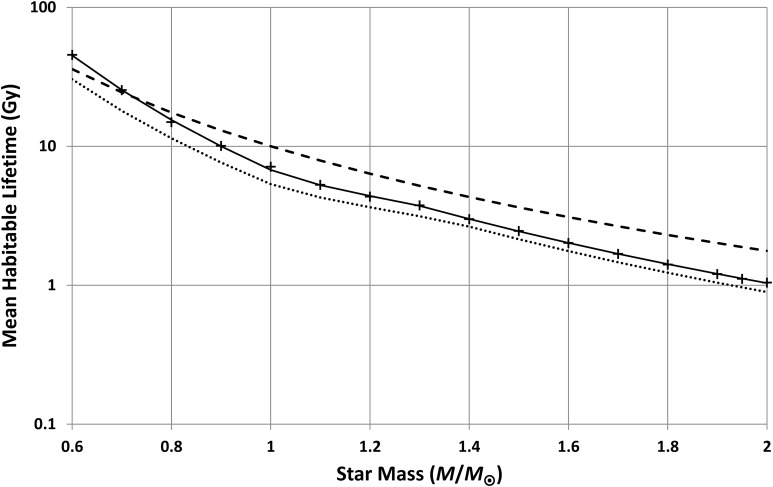
Mean habitable lifetime for planets, possessing intelligent observers, as a function of star mass. Crosses show the results produced by the models in this paper. The solid curve is a power-law fit to these models, while the dashed curve is the classic main sequence lifetime of 10(*M/M*_⊙_)^−2.5^. The dotted line shows the same calculations repeated for life, in general, rather than just intelligent life.

**FIG. 10.**
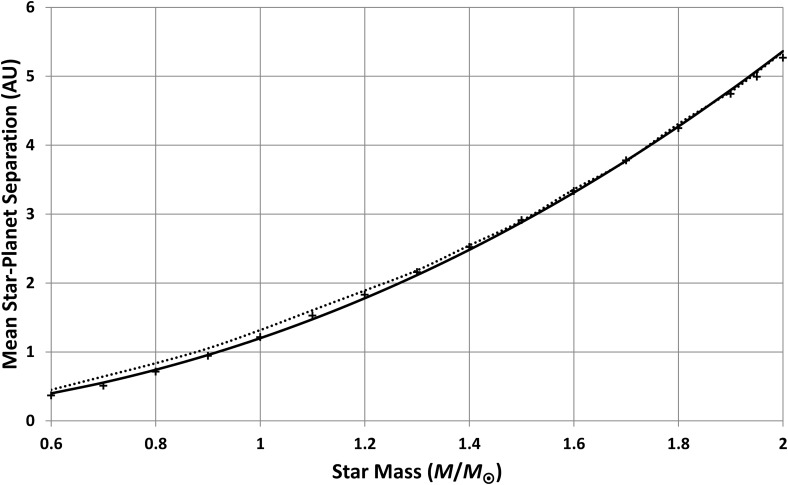
Mean star-planet separation for planets, possessing intelligent observers, as a function of star mass. Crosses show results from models in this paper. The solid curve is a power-law fit to these models. The dotted line shows the same calculations repeated for life, in general, rather than just intelligent life.

Repeating these calculations for other star masses produces Figs. 9 and 10. These results assume *n* = 4, but they are not changed greatly if *n* = 3 or 5. Note that the plots do not extend below 0.6 *M*_⊙_ because Girardi *et al.* (2000) (and other models the author is aware of) did not give the full main sequence evolution for these lower masses. This is probably because, for most purposes, there is little point in modeling stellar evolution over timescales much greater than the present age of the Universe. This omission is unfortunate as such models would have been useful here, but as will be shown later, this is not a fatal problem.

There are three distinct segments in Fig. 9: a smooth trend below 1 *M*_⊙_, a smooth trend above 1.3 *M*_⊙_, and a relatively low-gradient transition between these. A reasonable power-law fit is

\begin{align*}
\begin{split}& \bar \tau = { \rm{ }}6.76{ ( M / {M_ \odot } ) ^{ - 3.71}} \quad M   <   { \rm{ }}1.03 \ {M_ \odot } \\ & \bar \tau = { \rm{ }}6.39{ ( M / {M_ \odot } ) ^{ - 2.06}} \quad 1.03 \ {M_ \odot }  \le   M   \le   1.30 \ {M_ \odot } \\ & \bar \tau = 8.17{ ( M / {M_ \odot } ) ^{ - 2.98}} \ \ \ M   >  { \rm{ }}1.30 \ {M_ \odot }\end{split}
 \tag{13}\end{align*}

This can be compared to the classic order-of-magnitude estimate for main sequence lifetime (*e.g.*, see Hansen and Kawaler, 1994) that

\begin{align*}
\tau = { \rm{ }}10{ ( M / {M_ \odot } ) ^{ - 2.5}} \tag{14}
\end{align*}

Equations 13 and 14 are both shown in Fig. 9. Equation 14 overestimates the typical habitable lifetime for masses above 0.7 *M*_⊙_ and underestimates it below that threshold. Both equations will be used, in the next section, to demonstrate that results are not sensitive to plausible uncertainties in habitable lifetimes.

Similarly, the predicted star-planet separations, shown in Fig. 10, fit a power-law model of the form

\begin{align*}
\bar a = { \rm{ }}1.2{ ( M / {M_ \odot } ) ^{2.16}} \tag{15}
\end{align*}

for all masses considered.

## 6. The Trouble with Red Dwarfs

The preceding analyses provide the background needed for the key objective of this paper—an investigation of possible habitability problems for low-mass stars. Low-mass stars are both much more common and much longer-lived than larger stars, so if all else is equal, intelligent observers should nearly always find themselves orbiting small stars. But this expectation is contradicted by the observation that the Sun is not a red dwarf, so there may be a habitability problem associated with smaller stars. This section investigates this question by using the statistical methods developed above.

Star mass, *M*, is now the property of interest, and Eq. 4 becomes

\begin{align*}
p \left( {M / i} \right) \ { \rm{ }} = \ K \ q \left( M \right) p \left( M \right) \tau { \left( M \right) ^n} \tag{16}
\end{align*}

Here, *p*(*M*) is the probability that a randomly chosen, potentially habitable planet orbits a star of mass *M*. This probability is controlled by the frequency of such stars and by the frequency with which such stars have potentially habitable planets. The frequency of stars of a given mass is called the initial mass function (IMF) and has been the subject of much astronomical research and debate over many decades (*e.g.*, see Salpeter, 1955; Miller and Scalo, 1979; Kroupa, 2002; Chabrier, 2003, 2005), but there is still no final agreement on its exact form. This paper will therefore use two widely used distributions so that sensitivity to this factor can be properly illustrated. Firstly, Miller and Scalo (1979) gave

\begin{align*}
\begin{split} \xi (M) & = 0.20 \
{M^{-1.4}} \quad \quad 0.08 \ {M_\odot}  <  M  <  1 \ {M_\odot}
\\ & = 0.20 \ {M^{-2.5}} \quad \quad M   <   10 \ {M_\odot}
\end{split}
\tag{17} \end{align*}

while Chabrier (2005) gave

\begin{align*}
\begin{split} \xi (M)  & = (0.41 {/} M) {\rm \exp}  \left(- \frac {(\log(M) - \log 2)^2}
{0.605} \right)  \\ & { \qquad \qquad \quad \qquad \qquad \qquad 0.08 \ {M_\odot}} < M < 1 \ {M_\odot} \\
 & = 0.18 \ {M^{- 2.35}} \quad \quad \quad \quad \quad \quad \quad
\quad \quad \quad \ M < 10 \ {M_\odot}\end{split} \tag { 18 }
\end{align*}

**FIG. 11.**
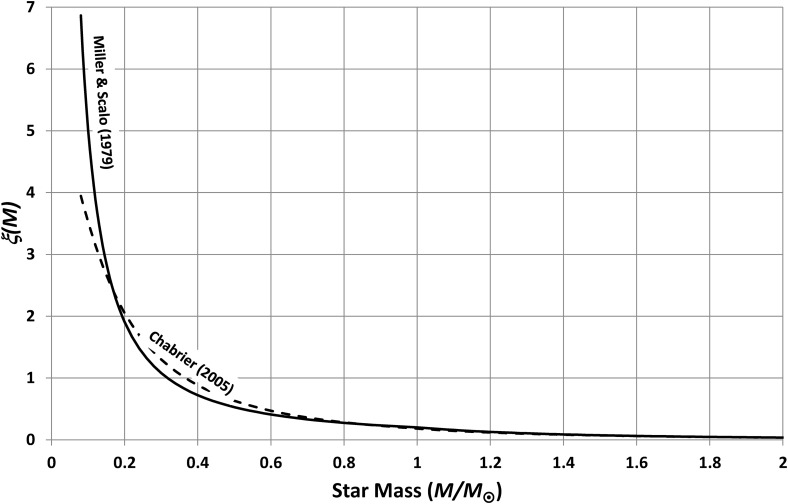
Initial mass functions used in this paper. These curves show how the numbers of stars vary with stellar mass and demonstrate that small stars are much more common than large stars.

The lower limit of 0.08 *M*_⊙_ corresponds to the lowest mass for hydrogen fusion. Note that these expressions have been modified slightly from their published form so that they give ξ(*M*)—rather than ξ(log *M*)—and so that they have integrals equal to unity. Equations 17 and 18 are plotted in Fig. 11, which shows that there is, in particular, a difference at low stellar masses where Eq. 17 gives a result almost 60% larger than Eq. 18. Nevertheless, both functions show a very rapid drop in frequency with mass; light stars are much more common than heavy ones.

Given these IMFs, the probability that a randomly chosen, potentially habitable planet orbits a star of mass *M* is

\begin{align*}
p \left( M \right) { \rm{ }} = f \left( M \right) \xi \left( M \right) \tag{19}
\end{align*}

where *f*(*M*) is the fraction of stars of mass *M* that have potentially habitable planets (normalized by the fraction of all stars that have potentially habitable planets). Equation 16 therefore becomes

\begin{align*}
p \left( {M / i} \right) { \rm{ }} = K \ q \left( M \right)  f \left( M \right) \xi \left( M \right) \tau { \left( M \right) ^n} \tag{20}
\end{align*}

**FIG. 12.**
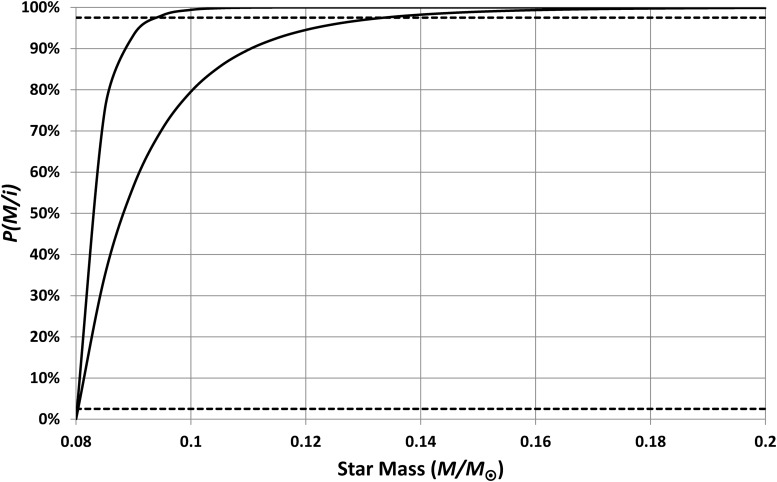
Cumulative probabilities for the masses of stars having inhabited planets. These curves assume that planets orbiting small stars are as common and as inhabitable as planets orbiting larger stars. The upper curve is the worst-case calculation, and the lower curve is the best-case calculation. Note that, even for the best-case scenario, these assumptions predict that 97.5% of all inhabited planets orbit stars smaller than 0.13 *M*_⊙_. Hence, these assumptions are not compatible with the observed large size for our Sun.

The simplest assumptions are then that *q*(*M*) and *f*(*M*) are both constant, that is, that all stars have equally habitable HZs and that the frequency of potentially habitable planets does not vary with star mass. Such assumptions do not give plausible results; this is shown by the cumulative probability curves of Fig. 12. The upper curve is the worst-case scenario (*i.e.*, the one that makes Earth most surprising) in which I have used Eqs. 13 and 17 with *n* = 6. The lower (best-case) curve uses Eqs. 14 and 18 along with *n* = 3. The cumulative probability for a typical inhabited planet should fall between 2.5% and 97.5% (the dashed lines) for 5% significance; hence, typical inhabited planets should orbit stars smaller than, at best, 0.13 *M*_⊙_.

To emphasize that this analysis makes Earth appear to be highly untypical, the results suggest that only one inhabited planet in 3 billion will orbit a star as large as the Sun (best-case). To further quantify the size of effect needed to make the Earth a typical inhabited planet, a simple assumption can be made that

**FIG. 13.**
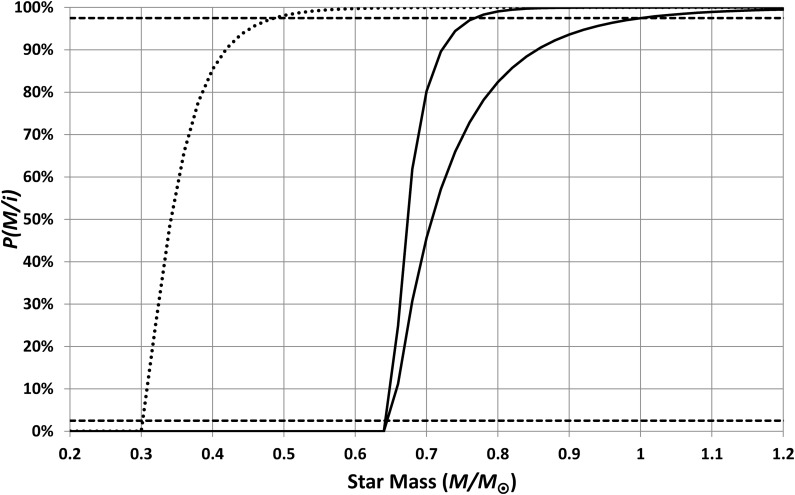
Cumulative probabilities for the masses of stars having inhabited planets. The solid curves assume that planets orbiting stars smaller than 0.65 *M*_⊙_ are uninhabitable. The upper curve is the worst-case calculation, and the lower curve is the best-case calculation. With this cutoff applied, the best-case curve is consistent with the observed size of our Sun. The dotted curve assumes that planets orbiting small stars are rendered uninhabitable by the effects of pre-main-sequence heating; this hypothesis is not compatible with the observed large size of our Sun.

\begin{align*}
q \left( M \right)   f \left( M \right) { \rm{ }} = { \rm{ }}0 \quad M   <  {M_{{ \min}}} \tag{21}
\end{align*}

where *M*_min_ is a stellar mass below which there are either no potentially inhabitable planets [*i.e.*, *f*(*M*) = 0] or below which planets are not habitable [*i.e.*, *q*(*M*) = 0]. Figure 13 shows the resulting cumulative probability distributions when the cutoff is set at 0.65 *M*_⊙_. This cutoff allows the best-case scenario to give a probability that *P*(*M* < 1 *M*_⊙_/*i*) = 97.5%; that is, this is the minimum cutoff that allows Earth to be a typical inhabited world. In summary, whatever the process is that makes planets orbiting small stars less habitable, it must have significant effects up to, at least, 0.65 *M*_⊙_.

It is instructive to look at possible mechanisms for poor habitability of planets orbiting low-mass stars, in light of the above result. A currently widely discussed mechanism is that low-mass stars take a relatively long time to reach the main sequence, and during that interval, HZ planets are exposed to very high temperatures that may strip them of their atmospheres. This issue was examined in detail by Luger and Barnes (2015), who concluded that this effect is very significant up to 0.3 *M*_⊙_ and may have effects up to around 0.6 *M*_⊙_. This can be modeled by assuming *q*(*M*) = 0 for *M* < 0.3 *M*_⊙_ and then ramps up to *q*(*M*) = 1 by *M* = 0.6 *M*_⊙_. The effect of this is shown by the dotted line in Fig. 13, which exceeds the 97.5% threshold for plausibility for *M* > 0.5 *M*_⊙_ hence suggesting that this mechanism is not sufficiently powerful to explain the surprisingly large size of our Sun. This result assumes *n* = 3, Eq. 13, and Eq. 17, but *n* > 3 makes the threshold for plausibility even lower, while the other choices for habitable lifetime (*i.e.*, Eq. 12) and IMF (*i.e.*, Eq. 17) make little difference at all. Results can be made closer to plausibility by having *q*(*M*) drop more rapidly below 0.6 *M*_⊙_, but even then they do not allow the Sun's mass to fall within the predicted 95% confidence range.

An alternative possibility is that the high X-ray, UV, and flare activity of young, small stars suppresses their habitability initially. However, this is only for a relatively short period compared to the habitable lifetimes shown in Fig. 9 [activity decreases even for low-mass stars after ∼1 Gy (Scalo *et al.*, 2007)]. Even if such processes prevent habitability for as much as 10 Gy, this does not account for the statistical anomaly (since Fig. 9 shows habitable lifetimes of small stars are significantly greater than 10 Gy) unless this early activity permanently renders orbiting worlds uninhabitable. In addition, Scalo *et al.* (2007) suggested that high activity is only serious for stars ∼0.36 *M*_⊙_ or smaller, and this is much less than the required cutoff of ∼0.65 *M*_⊙_. Thus, at present, radiation-dependent explanations for poor habitability of low-mass stars cannot explain the large mass of our Sun because they do not operate for long enough and cease operating at too low a mass cutoff. However, future work may show that the effects of radiation on habitability are more serious than currently believed.

Another possible explanation is that terrestrial planets are simply rare around smaller stars, that is, *f*(*M*) is low. However, the discovery of planets such as KOI-1843b [0.63 Earth-mass planet orbiting a 0.45 *M*_⊙_ star (Ofir and Dreizler, 2013)] or Kepler-42 d [0.13 *M*_⊙_ star with three small planets (Muirhead *et al.*, 2012)] indicates that, while such worlds may be less common around small stars, they are not rare by the factor of several billion needed to explain the statistical anomaly.

One final possibility is the oldest of the hypotheses but also the one that can be most thoroughly treated using the methods of this paper; planets orbiting in the close-in HZ of low-mass stars may be adversely affected by tidal locking, that is, tidal slowing of their rotation rates to the point where there is synchronous rotation so that a planet day equals a planet year. Lammer *et al.* (2009) and Scalo *et al.* (2007) reviewed this possibility and discussed how slow rotation may affect climate, magnetic-field strength, and exposure to radiation. However, the idea that planets orbiting red dwarfs may be adversely affected by such factors has been criticized by others (*e.g.*, Heath *et al.*, 1999; Yang *et al.*, 2014).

Fortunately, the methods developed in this paper allow the tidal-locking hypothesis to be tested without the uncertainties surrounding detailed atmospheric and/or geophysical modeling. We can simply assume tidal locking is detrimental to habitability for unspecified reasons and concentrate on investigating how tidal locking alters the statistical analysis given above.

Following the work of Gladman *et al.* (1996), the time to synchronous rotation is

**FIG. 14.**
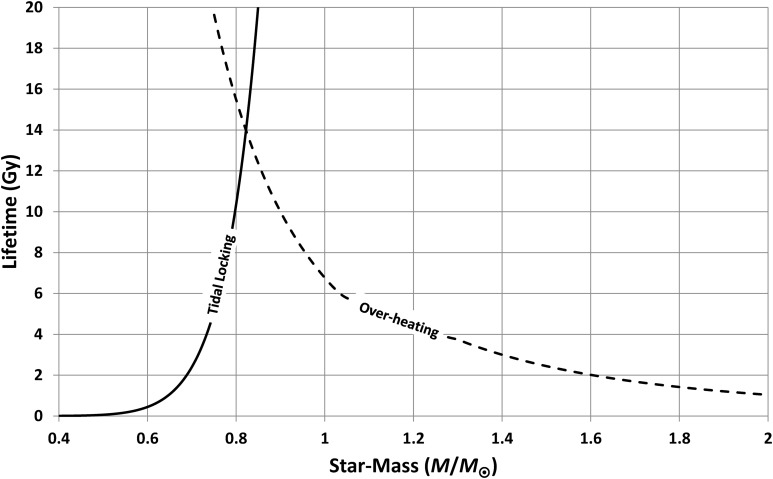
Time to tidal locking (Eq. 22) and time to overheating (Eq. 13). If both are catastrophes for habitability, then planets orbiting stars with masses around 0.84 *M*_⊙_ have the longest habitable lifetimes.

\begin{align*}
{ \tau _{{ \rm{despin}}}} = { \rm{ }} \left( { \omega C { \rm{ }}Q{ \rm{ }} / 3G{k_2}{R^5}} \right)  \left( {{a^6} / {M^2}} \right) \tag{22}
\end{align*}

where *ω* is the initial angular velocity of the planet, *C* is its moment of inertia, *Q* is the tidal quality factor (which controls energy dissipation to heat), *G* is Newton's constant of gravitation, *k*_2_ is the tidal Love number (a measure of the planet's rigidity), and *R* is the planet's radius. The values in the first bracket on the right-hand side can be assigned Earth values because, as discussed in the introduction, the starting assumption is that inhabited planets are likely to be Earth-like. With these parameters fixed, Eqs. 15 and 22 give the time to tidal locking shown in Fig. 14 (which also shows the habitable lifetime from Eq. 13, *i.e.*, time for overheating).

**FIG. 15.**
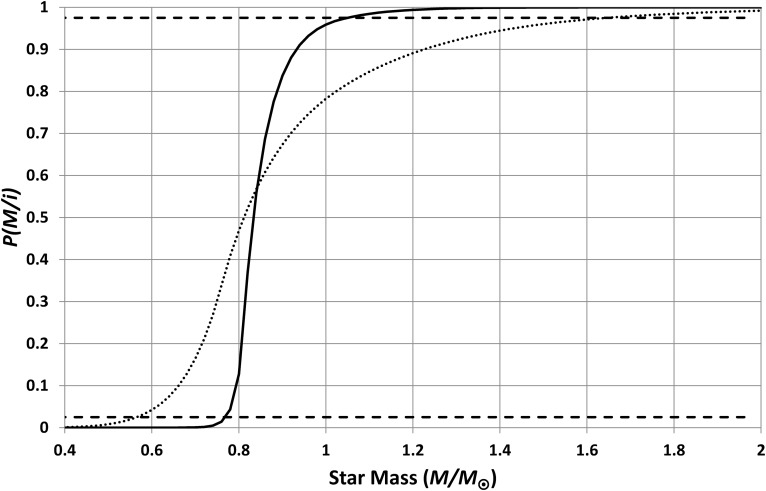
Cumulative probabilities for the masses of stars having inhabited planets. These curves assume that planets become uninhabitable when they become tidally locked or when they become overheated (whichever happens first). The solid line shows the calculation for planets inhabited by intelligent organisms, and the dotted line shows the calculation for life in general. These results are compatible with the observed mass of our Sun.

If habitability is detrimentally affected both by stellar-evolution-generated overheating and by tidal locking, then the habitable lifetime is the minimum of Eqs. 13 and 22; that is, lifetime is limited by tidal locking for planets orbiting stars smaller than 0.84 *M*_⊙_ and by star evolution for planets with stellar mass greater than this. Equations 17 and 20 then give the predicted star masses, for typical inhabited planets, shown in Fig. 15.

From Fig. 15, it is clear that the hypothesis that habitability is limited by both stellar evolution and by tidal locking predicts a range of inhabited stellar masses, which includes the solar mass; the 95% confidence range is 0.78 *M*_⊙_ < *M* < 1.04 *M*_⊙_. Hence, this hypothesis is supported by the analysis (strictly, the hypothesis is not rejected). Using Eq. 18 instead of Eq. 17 makes no significant difference to the results. Note that the probability shown in Fig. 15 is extremely small for *M* < 0.6 *M*_⊙_; hence, the fact that the power-law fits (Eqs. 13 and 15) are highly uncertain below this threshold is not important.

**FIG. 16.**
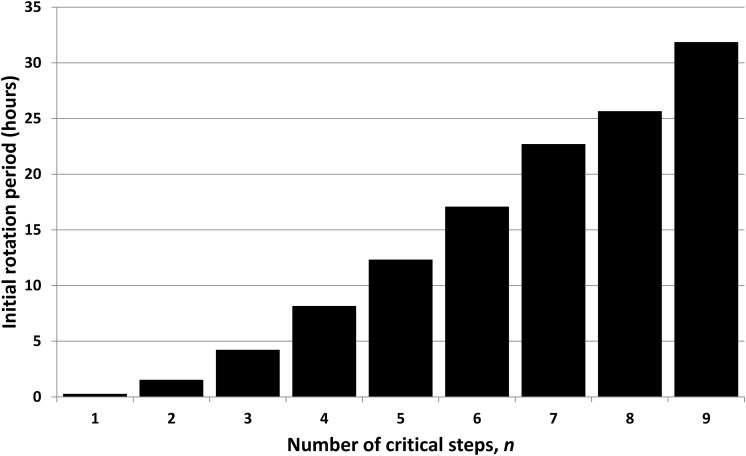
Minimum allowed initial rotation period of planets with intelligent observers for consistency with the hypothesis that habitability is limited by tidal locking. Actual periods of young terrestrial planets are of the order of a few hours; hence, *n* is unlikely to be larger than about 5 if the tidal locking hypothesis is correct.

However, the predicted distribution of stellar masses is dependent upon the choices for *n* (4 in Fig. 15) and the initial rotation rate (6 h in Fig. 15). Figure 16 shows how the minimum allowed initial rotation period increases with *n*. For any given *n*, shorter periods of rotation than those indicated result in 95% confidence ranges for stellar mass, which do not encompass the Sun's mass. Sensible initial periods (say less than 12 h) therefore imply *n* ≤ 5. Hence, either *n* is relatively small, or an alternative to the tidal locking hypothesis is needed to explain why our Sun is so large.

## 7. Life in General

The preceding sections have explicitly looked at the predicted properties of planets possessing intelligent observers. This allowed the resulting predictions to be directly compared with the known Earth properties to discern whether the various habitability hypotheses were supported. The resulting predictions may be useful for SETI with Fig. 15 indicating the range of star masses that are most promising.

However, this final section will relax the intelligent-life constraint and use Eq. 4 to predict distributions for planets that have passed only the first step (which is, plausibly, the origin of life itself). Thus, with *n* = 1, Eq. 4 gives the conditional probabilities given life, *p*(*x/L*), rather than conditional probabilities given intelligence, *p*(*x/i*). This section therefore recalculates distributions, using *n* = 1, to predict the star masses most likely to possess planets with life and the most-likely distances at which such planets orbit their stars.

The first step is to recalculate Eqs. 11–15 with *n* = 1. The resulting predictions of mean habitable lifetime and mean distance are shown as dotted lines in Figs. 9 and 10 with power-law fits

\begin{align*}
\begin{split}& \bar \tau = 5.34(M/{M_\odot})^{-3.4} \quad \ M  <  1.03 \ {M_\odot}
\\ & \bar \tau = 5.11(M/{M_\odot})^{- 1.86} \quad 1.03 \ {M_
\odot}  \le   M \le 1.36 \ {M_\odot}
\\ & \bar \tau = 7.34(M/{M_\odot})^{- 3.04} \quad 1.36 \ {M_\odot} <
M \end{split}
 \tag{23}
\end{align*}

and

\begin{align*}
\bar a = { \rm{ }}1.3{ ( M / {M_ \odot } ) ^{2.03}} \tag{24}
\end{align*}

Generally, the changes from the “intelligent observer” results are small, for distance, but a reduction in mean habitable lifetime results from the fact that the range of habitable lifetimes, compatible with the emergence of life, will include shorter lifetimes than the range needed for intelligent life. Hence, the average drops.

With these new power-law models for the expected lifetime and separation, the cumulative probability can be recalculated, using *n* = 1, to give the dotted line shown in Fig. 15. This has a 95% confidence range of 0.57 *M*_⊙_ < *M* < 1.64 *M*_⊙_ which is, as expected, broader than the range for intelligent life.

## 8. Discussion

The results of this paper should be treated as provisional since there are many caveats. Nevertheless, the techniques have given useful insights concerning the most promising places to look for Earth-like life (*i.e.*, life on the stellar-heated surface of a planet).

The first caveat is that the approach is inappropriate if we are considering habitats, such as the subsurface oceans of icy moons, that are very different from Earth. Secondly, as with any statistical technique, the approach attempts to reject, rather than accept, hypotheses, so it is always possible that another hypothesis exists that is as good, or better, than the one under consideration. In the specific case of the results obtained in this paper, there may be other hypotheses that account equally well for the poor habitability of low-mass stars. However, other explanations will need to have a broadly similar effect to satisfy the requirement that they “explain Earth” (*e.g.*, any low-mass habitability problem should cause difficulties for stellar masses <0.65 *M*_⊙_), so the resulting predictions of “best star mass” are likely to be similar.

Another caveat is that the results concerning “life in general” have assumed that the origin of life is the first step in the *n*-step model. This may not be correct. Given our poorly constrained knowledge of the timing for the origin of life, it is possible that life actually arises quickly and the first “hard” step is something later (*e.g.*, photosynthesis). Alternatively, there may be a pre-life “hard” step such as the need for an unusual combination of geological circumstances that allow concentration of key prebiotic chemical compounds. The predictions in the preceding section therefore concern the distribution of planets that have taken the first step, whatever that is. However, it is not unreasonable to suggest that this may be the origin of life itself.

A final caveat is that the results are completely dependent upon Carter's (1983) *n*-step model for the emergence of intelligence. This author, however, finds his arguments compelling, and interested readers are advised to read the works of Carter (1983) and Watson (2008) if they require further reassurance.

A more specific issue is that, even if the conclusion is accepted that tidal locking is the cause of low-star-mass habitability problems, the analysis cannot tell us why this is the case. Of course, this is also a strength of the technique in that the conclusion is not dependent upon process details. Nevertheless, the techniques cannot tell us if poor habitability is caused by climatic issues (*e.g.*, collapse of the planet's atmosphere on the point opposite the star), magnetic field issues (*e.g.*, insufficient field-strength to prevent loss of atmosphere through sputtering), or something not previously considered in any study (*e.g.*, the inability of a tidally locked planet to have a dynamically stable moon). Thus, the results of this paper suggest that further work on the consequences of tidal locking would be worthwhile.

Despite all these issues and caveats, the methods presented in this paper have allowed habitability hypotheses to be challenged in a new way, and they have allowed several predictions for properties of Earth-like habitats. The approach therefore provides useful new insights into where we should look for life beyond Earth.

This paper has also highlighted how important it is, for astrobiology, that we get better estimates of the timing of the origin of life on Earth. Clearly, this would improve estimates of *n*, but more fundamentally, it could also impact greatly our estimates of the likelihood of finding life beyond Earth. The Carter (1983) model predicts that life will be very rare (and intelligent life much rarer still), and this model is supported by the fact that the time taken for life to emerge on Earth appears to be of a similar duration to the time left for life after the emergence of intelligence. However, if evidence for a much earlier appearance of life emerges so that this coincidence breaks down, the conclusion will either be that the Carter model is invalid or that life emerges easily and is not the first step in the *n*-step process leading to intelligence. Either way, life will be much more common than the Carter model suggests.

## 9. Conclusions

(1) Equation 4 can be used to estimate pdfs for properties of planets possessing intelligent observers. If the resulting 95% confidence range does not encompass Earth's value, this may indicate issues with the underlying habitability assumptions.
(2) This methodology allows models of HZ location to be tested.
(3) The outer edge of Earth's current habitability zone is bounded by 1.18 AU < *a*_o0_ < 1.50 ± 0.14 AU.
(4) If all HZs are equally habitable, then the 95% confidence range, for the masses of stars with planets hosting intelligent observers, only extends to 0.13 *M*_⊙_. Hence, our Sun is surprisingly large unless there is a mechanism that suppresses the habitability of planets orbiting low-mass stars.
(5) For Earth to be a typical inhabited planet, there must be very substantial suppression of habitability for stars of mass below ∼0.65 *M*_⊙_.
(6) Conclusion 5 is difficult to reconcile with explanations based upon the poor radiation environment in the HZ of smaller stars.
(7) Conclusion 5 is difficult to reconcile with explanations based upon a paucity of suitable planets orbiting smaller stars.
(8) Conclusion 5 is compatible with explanations that assume the HZs of smaller stars are poor habitats because of tidal locking.
(9) If tidal locking is the key process reducing the habitability of planets orbiting small stars: (a) The most promising targets for SETI are planets orbiting stars of mass 0.78 *M*_⊙_ < *M* < 1.04 *M*_⊙_. (b) The most promising targets for searching for life in general are planets orbiting stars of mass 0.57 *M*_⊙_ < *M* < 1.64 *M*_⊙_. (c) There are unlikely to be more than *n* = 5 critical evolutionary steps required for the emergence of intelligence.

## Abbreviations Used

Ga: billions of years ago (an age)
Gy: billions of years (a duration)
HZ: habitable zone
IMF: initial mass function
pdf: probability density function
